# First study on the outcomes of biliopancreatic diversion with duodenal switch in Chinese patients with obesity

**DOI:** 10.3389/fsurg.2022.934434

**Published:** 2023-01-06

**Authors:** Lun Wang, Zheng Zhang, Zeyu Wang, Tao Jiang

**Affiliations:** Department of Bariatric and Metabolic Surgery, China-Japan Union Hospital of Jilin University, Changchun, China

**Keywords:** biliopancreatic diversion, duodenal switch, obesity, weight loss, outcomes

## Abstract

**Background:**

Biliopancreatic diversion with duodenal switch (BPD-DS) is a bariatric procedure used in the treatment of obesity and related metabolic disorders. However, to date, the data on BPD-DS among Chinese patients with obesity is completely lacking.

**Objective:**

This is the first study to evaluate the safety and efficacy of BPD-DS in the treatment of Chinese patients with obesity.

**Methods:**

Data from 12 patients undergoing BPD-DS between September 2019 and March 2020 were analyzed retrospectively to evaluate complications, weight loss, comorbidity resolution, and nutritional status.

**Results:**

All patients completed the 1-year follow-up. There was no conversion to laparotomy or death. Mean operative time was 257.08 ± 29.27 min. The median length of stay was 7 days (ranging from 4–38 days). Complications occurred in three patients. The 1-year body mass index was 25.13 ± 4.71 kg/m^2^ with a mean excess weight loss of 100.11 ± 33.29% and a mean total weight loss of 43.22 ± 7.71%. Remission was achieved in 100% (7/7 cases) for type 2 diabetes, in 83.3% (10/12 cases) for hypertension, and in 62.5% (5/8 cases) for hyperuricemia. At 1 year after BPD-DS, the prevalence of albumin deficiency was 16.7%, for iron deficiency was 16.7%, and for zinc deficiency was 50%. There were 8.3% of the patients who were anemic. High deficiency rates for vitamins were presented mainly in vitamin A and vitamin E: vitamin A in 58.3% of the patients and vitamin E in 50% of the patients. The prevalence of asymptomatic gallstones increased significantly from 0% preoperatively to 41.7% postoperatively.

**Conclusion:**

BPD-DS had excellent weight loss and resolution of comorbidities among Chinese patients with obesity. However, high rates of nutritional deficiencies and complications were found after BPD-DS, especially for vitamin A and vitamin E.

## Introduction

The prevalence of obesity is increasing dramatically in either developed or developing countries. It is estimated that the number of globally obese adults will reach 573 million individuals by 2030 (1). Obesity significantly increases the risks of type 2 diabetes, hypertension, hyperuricemia, dyslipidemia, cardiovascular disease, and even some cancers, which pose a serious threat to human health (2, 3).

Bariatric surgery has been proven to be more effective in the treatment of obesity and obesity-related metabolic disorders when compared with conventional therapy (4–6). Among the most commonly performed bariatric operations, biliopancreatic diversion with duodenal switch (BPD-DS) is considered to be the most effective procedure in terms of weight loss and comorbidity remission (7–9). Despite having been used in the treatment of obesity for about 30 years since Marceau et al. (10) first described this procedure in 1993, two main criticisms of BPD-DS are only a few bariatric centers with a regular practice of this procedure, and the lack of reports originating from surgeons in non-Western countries. Until now, there are no known studies reporting the outcomes of BPD-DS in the treatment of Chinese patients with obesity. Consequently, this study aims to estimate the efficacy and safety of BPD-DS among Chinese patients with obesity.

## Materials and methods

### Patients

From September 2019 to March 2020, a total of twelve patients who underwent laparoscopic or robotic BPD-DS as a primary procedure for obesity at a university-affiliated bariatric center were included in this study.

### Definitions

The Dindo–Clavien classification was used to classify the severity of complications (11). Nutritional deficiencies were defined as a serum level below the lower limit of the normal range. Excess weight loss (EWL) = (preoperative weight − postoperative weight)/(preoperative weight − ideal weight), ideal weight is calculated as ideal body mass index (BMI) times height in meters squared, ideal BMI = 23 kg/m^2^. Total weight loss (TWL) = (preoperative weight −postoperative weight)/preoperative weight.

### Operative technique

We performed BPD-DS by the Da Vinci Xi® surgical system or laparoscopy step-by-step. First, starting from the ileocecal junction, a common channel of 100 cm and an alimentary limb of 200 cm were measured in a retrograde manner and marked with sutures, respectively. Second, the gastrocolic ligament was dissected along the greater curvature of the stomach, and then a sleeve gastrectomy was performed over a 36-Fr bougie using a linear stapler from 3 cm proximal to the pylorus to the angle of His. Third, the duodenum was dissected and transected at 2 cm distal from the pylorus. An end-to-side duodenoileal anastomosis was performed between the proximal duodenal stump and the ileal at 300 cm proximal from the ileocecal valve. Fourth, the small intestinal was transected at 2 cm proximal from the duodenoileal anastomosis with a linear stapler, and then we created an ileo-ileostomy between the biliopancreatic limb and ileum at 100 cm proximal from the ileocecal valve. Fifth, the mesenteric defect was routinely closed, while the Petersen space was not routinely closed. Lastly, the reinforcement was performed by stitching the gastric staple line and omentum using a continuous suture.

### Nutritional supplementation

All patients were recommended to take nutritional supplements after tolerating a semi-liquid diet. The daily intakes of each patient were as follows: Vitamin B12 100 µg; one pill of Calcium Carbonate And Vitamin D3 Chewable Tablets® (total nutrients: Calcium Carbonate 750 mg and vitamin D3 60 IU); one pill of Compound Vitamin B Tablets® (total nutrients: vitamin B_1_ 1.5 mg, vitamin B_2_ 1.7 mg and vitamin B_6_ 2 mg); one pill of Vitamin AD Soft Capsules® (total nutrients: vitamin A 10,000 IU and vitamin D 1,000 IU); one pill of Centrum® (total nutrients: vitamin A 5,000 IU, vitamin D 400 IU, vitamin E 30 IU, vitamin B_1_ 1.5 mg, vitamin B_2_ 1.7 mg, vitamin B_6_ 2 mg, vitamin B_12_ 6 µg, vitamin C 60 mg, vitamin K_1_ 25 µg, folic acid 400 g, kalium 40 mg, calcium 162 mg, magnesium 100 mg, iron 18 mg, zinc 15 mg, phosphorus 125 mg and so on). We also advice a minimal protein intake of 60 g per day and up to 1.5 g/kg of ideal body weight. These supplements were individually adjusted on the basis of blood studies obtained during follow-up.

### Statistical analysis

SPSS 22.0 was used for statistical analysis. Continuous variables were presented as mean ± standard deviation and categorical variables were presented as number (percentage). We analyzed the changes between preoperative and postoperative indexes by the paired-sample Student's *t*-test (normality data) or Mann–Whitney *U* test (skewed data) or Chi-square test (categorical data), as appropriate. A *P*-value of <0.05 was considered statistically significant.

## Results

### Demographic data

A total of 12 patients who underwent laparoscopic or robotic BPD-DS between September 2019 and March 2020 were included in this study, and the overall follow-up rate was 100%. The patient demographic data are summarized in Table 1.

**Table 1:** Patient demographics.

Factor	All patients (*n* = 12)
Gender (male/female)	8/4
Mean age (years)	34.00 ± 9.92
Preoperative body weight (kg)	134.92 ± 22.90
Preoperative BMI (kg/m^2^)	44.93 ± 9.33
Previous upper abdominal surgery	0
Obesity-related comorbidity	
Type 2 diabetes	7 (58.3%)
Hypertension	12 (100%)
Hyperuricemia	8 (66.7%)

### Operative outcomes and complications

There was no conversion to laparotomy or death in our series. Mean operative time was 257.08 ± 29.27 min. Median length of postoperative hospital stay was 7 days (range 4–38 days). In total, complications occurred in three patients, including one abdominal bleeding (Dindo–Clavien grade II), one duodenal-ileal anastomotic leakage (Dindo–Clavien grade IIIa), and one small intestine obstruction (Dindo–Clavien grade IIIb). The patient, diagnosed with small intestine obstruction, was readmitted and reoperated because of severe intestinal obstructive symptoms and malnutrition. After reversal, her intestinal obstructive symptoms remarkably improved and her nutritional status returned to normal. The remaining two patients with complications were cured successfully by the conservative treatment. In addition, the prevalence of asymptomatic gallstones increased significantly from 0% preoperatively to 41.7% postoperatively.

Gastrointestinal side effects at 1-year after BPD-DS were: 7 out of 12 patients experienced a stool frequency of three to five times per day; 4 patients had a stool frequency less than three times per day;1 patient had a stool frequency of 8 times per day; 2 out of 12 patients had occasional reflux symptoms; and no patients experienced nausea and vomiting.

### Weight loss and comorbidities remission

**Figure 1:**
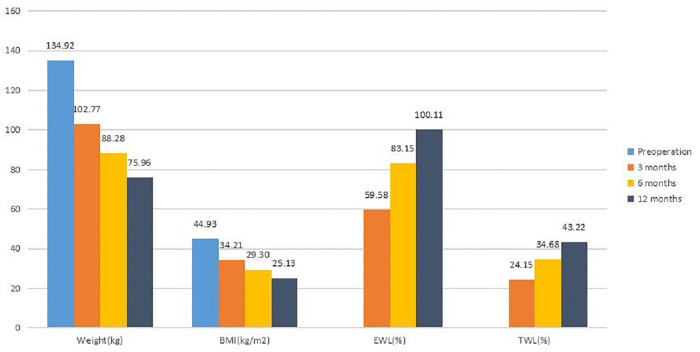
The changes of weight, BMI, EWL and TWL after BPD-DS.

The weight loss data are shown in Figure 1. In terms of weight and BMI, a significant difference was seen between preoperation and 3 months, 3 and 6 months, and 6 and 12 months (*p* < 0.05).

For EWL and TWL, a significant difference was observed between 3 and 6 months and 6 and 12 months (*p *< 0.05). The results indicated that patients gained sustained weight loss after BPD-DS. Of the 12 patients with hypertension, 10 patients achieved a level of blood pressure less than 140/90 mmHg and they no longer required medical treatment. All seven diabetic patients returned to normal levels of HBA1c and fasting plasma glucose and off all medications. Also, five of the eight patients with hyperuricemia achieved normal levels of uric acid without any medical treatment.

### Nutritional outcomes

To evaluate the nutritional condition, we compared the most recent nutritional markers with those obtained prior to surgery. Nutritional data are shown in Table 2. The mean levels of prealbumin, hemoglobin, hematocrit, vitamin A, vitamin E, folic acid, zinc, and iron were significantly decreased at 1 year after BPD-DS compared with those obtained prior to surgery. However, the mean levels of sodium and chloride were significantly increased after surgery. The deficiency rates of vitamin E and zinc were significantly increased at 1 year after BPD-DS compared with before surgery. At 1 year after BPD-DS, the level of albumin was below normal in one patient (albumin level ≥30 but <34.9 g/L), and one patient had severe hypoalbuminemia (albumin level <30 g/L). One patient had anemia (hemoglobin level <100 g/L). Of note, the hemoglobin level was significantly lower at 1 year after BPD-DS than before surgery, and this was associated with a significant decrease in the mean iron level (*r* = 0.753, *p *= 0.005).

**Table 2:** Changes of nutrients’ serum levels at 1 year after BPD-DS (*n* = 12).

	Normal range	Nutrients level		Deficiency (n)		P1	P2
		Baseline	1 year	Baseline	1 year		
Total protein (g/L)	62–83	72.40 ± 6.00	67.70 ± 6.54	1	2	0.058	1.000
Prealbumin (mg/L)	180–390	254.97 ± 38.54	213.15 ± 63.87	0	3	**0**.**049**	0.217
Albumin (g/L)	35–52	42.19 ± 2.55	39.67 ± 4.37	0	2	0.133	0.478
Hemoglobin (g/L)	110–150	151.35 ± 17.50	133.09 ± 14.70	0	1	**0**.**008**	1.000
Hematocrit (L/L)	0.37–0.48	44.47 ± 3.74	39.86 ± 4.04	0	2	**0**.**009**	0.478
Vitamin A (µg/ml)	0.38–0.98	0.46 ± 0.14	0.28 ± 0.12	3	7	**0**.**001**	0.107
Vitamin B12 (pg/ml)	180–916	400.86 ± 204.93	509.71 ± 303.58	0	2	0.345	0.478
Vitamin D (ng/ml)	3–29	12.43 ± 5.15	9.95 ± 5.76	0	0	0.256	—
Vitamin E (µg/ml)	5.7–19.9	12.23 ± 4.34	7.16 ± 2.57	0	6	**0**.**008**	**0**.**014**
Folic acid (ng/ml)	≥3.2	17.43 ± 8.33	8.77 ± 4.49	0	1	**0**.**003**	1.000
Sodium (mmol/L)	136–145	137.58 ± 3.06	140.34 ± 2.89	3	1	**0**.**021**	0.590
Kalium (mmol/L)	3.5–5.2	4.04 ± 0.38	3.98 ± 0.48	1	1	0.677	1.000
Calcium (mmol/L)	2.1–2.65	2.31 ± 0.15	2.34 ± 0.12	0	1	0.493	1.000
Chloride (mmol/L)	96–108	100.75 ± 3.92	105.79 ± 4.39	1	1	**0**.**001**	1.000
Phosphorus (µmol/L)	0.81–1.45	1.10 ± 0.23	1.21 ± 0.18	2	0	0.239	0.478
Magnesium (mmol/L)	0.8–1.00	0.82 ± 0.05	0.87 ± 0.10	2	3	0.124	1.000
Iron (µmol/L)	8.9–32.3	16.74 ± 4.09	13.12 ± 3.85	0	2	**0**.**021**	0.478
Zinc (µmol/L)	11.1–19.5	13.57 ± 1.53	11.17 ± 1.90	0	6	**0**.**009**	**0**.**014**

P1 means the comparison of nutrients level at baseline and 1 year after BPD-DS; P2 means the comparison for the rate of nutrients deficiency at baseline and 1 year after BPD-DS. Bold italic values indicate *P* < 0.05.

## Discussion

In China, the two most commonly performed bariatric procedures are sleeve gastrectomy (SG) and Roux-en-Y gastric bypass (RYGB), which account for nearly 98% of total bariatric operations. However, both SG and RYGB were reported to have higher rates of weight regain (12–14). Weight regain was also found for both RYGB and SG in our center. Consequently, we began to take an interest in BPD-DS because it enabled patients to achieve long-term weight loss (15, 16).

This is, to our knowledge, the first study to report the outcomes of BPD-DS in the treatment of Chinese patients with obesity. Our findings showed excellent outcomes following BPD-DS in terms of weight loss and comorbidity remission. Our study had an average preoperative BMI of 44.93 kg/m^2^. To date, there is not any consensus on whether or not BPD-DS should be performed in patients with a BMI less than 50 kg/m^2^. Nonetheless, this topic has been discussed in the literature, suggesting that BPD-DS could be considered in patients with a BMI < 50 kg/m^2^. In non-super-obese patients, BPD-DS was very efficient in terms of weight loss and patient satisfaction. The long-term risks of malnutrition and nutritional deficiencies exist but are usually manageable with medical treatment and only seldom require reoperation. In 2010, Biertho et al. (17) explored whether or not BPD-DS was indicated for patients with BMI less than 50 kg/m^2^. They found that the patients’ weight loss remained stable for 15 years after the operation and represented 76% of the initial excess weight. Malnutrition required readmission in 4.3% of cases and surgical revision in 1.5%. The same question was addressed by Wang et al. (18) in 2022, which evaluated the safety of BPD-DS in patients with a BMI less than 50 kg/m^2^. The authors demonstrated that BPD-DS may be considered in patients with BMI < 50 kg/m^2^. Careful patient selection and proper counseling of the risks and benefits are necessary.

In this study, the 1-year EWL was 100.11%. Our result is similar to the study reported by Cloutier et al. (19) who also found excellent outcomes in weight loss with an EWL of 93.4% at 1 year after BPD-DS. However, the weight loss outcomes of BPD-DS in other studies were inferior to our results (20–22), perhaps because we had a lower preoperative BMI and used a smaller bougie sizing tube.

Our study showed that the remission rate of type 2 diabetes (HBA1c < 6.0%) was 100% at 1 year after BPD-DS. This is in accordance with the results reported from other published studies on BPD-DS showing a diabetic remission rate of 100% (19, 23, 24). Other studies also showed a very high diabetic remission rate, but slightly lower than our results (25–27). Interestingly, our study showed that the levels of HBA1c and fasting plasma glucose in non-diabetic patients were also significantly decreased after BPD-DS.

Our study showed a high average length of stay-7 days. The reason for this difference is that, unlike SG and RYGB, BPD-DS is a more malabsorptive bariatric operation. It is quite possible that patients have trouble taking in adequate liquids after BPD-DS, resulting in dehydration. In addition, patients may also experience food intolerance after BPD-DS. In order to avoid dehydration due to insufficient liquid intake after BPD-DS, all patients were scheduled for intravenous hydration in our center in the first 3 postoperative days. We gradually decrease the amount of intravenous hydration and increase the intake of liquid diet at days 4–6 postoperatively. Usually, patients are informed of their discharged from the hospital at days 6–7 postoperatively. A similar treatment protocol was also reported in the study by Cottam et al. (28).

There were three complications in this series, including one bleeding, one leakage, and one small bowel obstruction. The intestinal obstruction occurred at the ileal-ileal anastomosis due to the fact that the mesentery was adhered and angulated with the anastomosis. The patient with small bowel obstruction had to be reversed because of severe nausea, vomiting, and malnutrition. These results were similar to those of other studies (17, 29, 30). No internal hernia occurred in our study, which was lower as compared with the studies by Pereira et al. (31) and Strain et al. (26). Although all patients were advised to take a standard dose of ursodeoxycholic acid after BPD-DS, the 1-year incidence of asymptomatic gallstones was up to 41.7%. There is not any consensus on whether or not prophylactic cholecystectomy should be performed in patients with normal gallbladders during BPD-DS. Prophylactic cholecystectomy could bring some benefits, such as avoiding gallstone formation and stone-related complications and reducing costs and hospitalization related to additional surgery. Nonetheless, the routine application of prophylactic cholecystectomy can also bring many disadvantages, which have been discussed in the literature, suggesting that prophylactic cholecystectomy should not be recommended routinely in BPD-DS patients with normal gallbladders. In 2018, a meta-analysis by Tustumi et al. (32) evaluated biliary complications concomitant with bariatric surgery and after surgery. They found higher rates of complications with concomitant cholecystectomy. Bardaro et al. (33) assessed the need for cholecystectomy in the postoperative period in BPD-DS patients. They found that only 19 (8.7%) had their gallbladder removed postoperatively, with an average follow-up of 30 months, and cholecystitis was rare. Sucandy et al. (34) evaluated the incidence of biliary complications in patients who underwent BPD-DS without simultaneous cholecystectomy. The authors found that the risks of developing biliary events were comparable to those in the general population.

In this study, vitamin deficiency presented mainly in vitamin A and vitamin E, and the mean levels of vitamin A and vitamin E were significantly decreased at 1 year after BPD-DS compared with those obtained prior to surgery, which were in accordance with the study by van Vollenstee et al. (35). Topart et al. (27) reported that 14.5% of the patients had albumin level below 35 g/L and 47.8% had prealbumin level below <200 mg/L. These were similar to the ones reported from our study. The deficiency rate of zinc was 50% in the present series which was higher than the result reported from Magee et al. (36). Strain et al. (37) reported the changes of various nutritional markers in the 9 years after BPD-DS. They found that the deficiency rates for many nutritional markers were much higher at 5- and 9-year follow-up compared with the 1-year nutritional outcomes. Consequently, with the extension of follow-up time, we still need to strengthen follow-up so as to timely modify nutrient supplementation.

Our study showed that the mean levels of sodium and chloride were significantly increased at 1 year after BPD-DS compared with those obtained prior to surgery but significantly decreased in hematocrit, perhaps because of insufficient fluid intake after surgery. The hemoglobin level decreased significantly at 1 year after BPD-DS, which was in accordance with the study by Moon et al. (24). However, Moon et al. reported a significant increase in hemoglobin level at 24 months compared with those obtained at 12 months. Anemia in the present series was found in 8.3% of all patients, which was similar to the report by Topart et al. (38), but higher than the study by Ballesteros-Pomar et al. (39).

This is the first study to report the outcomes of BPD-DS in the treatment of Chinese patients with obesity. However, the study has some limitations. On the one hand, it was a retrospective study with short-term following up. On the other hand, this study had a small sample size. Randomized controlled trials using large sample sizes are required for further study.

## Conclusion

BPD-DS had excellent weight loss and resolution of comorbidities among Chinese patients with obesity. However, high rates of nutritional deficiencies and complications were found after BPD-DS, especially for vitamin A and vitamin E.

## Data Availability

The raw data supporting the conclusions of this article will be made available by the authors, without undue reservation.

## References

[1] Kelly T Yang W Chen CS Reynolds K He J. Global burden of obesity in 2005 and projections to 2030. Int J Obes (Lond). (2008) 32(9):1431–7. 10.1038/ijo.2008.102 18607383

[2] Haslam DW James WPT. Obesity. Lancet. (2005) 366(9492):1197–209. 10.1016/s0140-6736(05)67483-1 16198769

[3] Hubert HB Feinleib M McNamara PM Castelli WP. Obesity as an independent risk factor for cardiovascular disease: a 26-year follow-up of participants in the Framingham Heart Study. Circulation. (1983) 67(5):968–77. 10.1161/01.cir.67.5.968 6219830

[4] Miras AD le Roux CW. Metabolic surgery versus conventional therapy in type 2 diabetes. Lancet. (2021) 397(10271):256–7. 10.1016/s0140-6736(21)00129-x 33485432

[5] Park JY Heo Y Kim YJ Park JM Kim SM Park DJ Long-term effect of bariatric surgery versus conventional therapy in obese Korean patients: a multicenter retrospective cohort study. Ann Surg Treat Res. (2019) 96(6):283–9. 10.4174/astr.2019.96.6.283 31183332 PMC6543048

[6] Kashyap SR Bhatt DL Wolski K Watanabe RM Abdul-Ghani M Abood B Metabolic effects of bariatric surgery in patients with moderate obesity and type 2 diabetes: analysis of a randomized control trial comparing surgery with intensive medical treatment. Diabetes Care. (2013) 36(8):2175–82. 10.2337/dc12-1596 23439632 PMC3714483

[7] Buchwald H Estok R Fahrbach K Banel D Jensen MD Pories WJ Weight and type 2 diabetes after bariatric surgery: systematic review and meta-analysis. Am J Med. (2009) 122(3):248–0056.e5. 10.1016/j.amjmed.2008.09.041 19272486

[8] Skogar ML Sundbom M. Weight loss and effect on co-morbidities in the long-term after duodenal switch and gastric bypass: a population-based cohort study. Surg Obes Relat Dis. (2020) 16(1):17–23. 10.1016/j.soard.2019.09.077 31711945

[9] Finno P Osorio J García-Ruiz-de-Gordejuela A Casajoana A Sorribas M Admella V Single versus double-anastomosis duodenal switch: single-site comparative cohort study in 440 consecutive patients. Obes Surg. (2020) 30(9):3309–16. 10.1007/s11695-020-04566-5 32240495

[10] Marceau B Bourque P Hould S. Biliopancreatic diversion with a new type of gastrectomy. Obes Surg. (1993) 3(1):29–35. 10.1381/096089293765559728 10757900

[11] Schlegel A van Reeven M Croome K Parente A Dolcet A Widmer J A multicentre outcome analysis to define global benchmarks for donation after circulatory death liver transplantation. J Hepatol. (2022) 76(2):371–82. 10.1016/j.jhep.2021.10.004 34655663

[12] Felsenreich DM Langer FB Kefurt R Panhofer P Schermann M Beckerhinn P Weight loss, weight regain, and conversions to Roux-en-Y gastric bypass: 10-year results of laparoscopic sleeve gastrectomy. Surg Obes Relat Dis. (2016) 12(9):1655–62. 10.1016/j.soard.2016.02.021 27317599

[13] Clapp B Wynn M Martyn C Foster C O'Dell M Tyroch A. Long term (7 or more years) outcomes of the sleeve gastrectomy: a meta-analysis. Surg Obes Relat Dis. (2018) 14(6):741–7. 10.1016/j.soard.2018.02.027 29625744

[14] Hawkins RB Mehaffey JH McMurry TL Kirby J Malin SK Schirmer B Clinical significance of failure to lose weight 10 years after Roux-en-Y gastric bypass. Surg Obes Relat Dis. (2017) 13(10):1710–6. 10.1016/j.soard.2017.08.004 28919184 PMC5761744

[15] Hess DS Hess DW Oakley RS. The biliopancreatic diversion with the duodenal switch: results beyond 10 years. Obes Surg. (2005) 15(3):408–16. 10.1381/0960892053576695 15826478

[16] Bolckmans R Himpens J. Long-term (>10 yrs) outcome of the laparoscopic biliopancreatic diversion with duodenal switch. Ann Surg. (2016) 264(6):1029–37. 10.1097/SLA.0000000000001622 26764870

[17] Biertho L Biron S Hould FS Lebel S Marceau S Marceau P. Is biliopancreatic diversion with duodenal switch indicated for patients with body mass index <50 kg/m^2^? Surg Obes Relat Dis. (2010) 6(5):508–14. 10.1016/j.soard.2010.03.285 20627706

[18] Wang A Nimeri A Genz M Feimster J Thompson K Abdurakhmanov A Safety of biliopancreatic diversion with duodenal switch in patients with body mass index less than 50 kg/m(2). Surg Endosc. (2022). 10.1007/s00464-022-09483-5. [Epub ahead of print] 35922604

[19] Cloutier A Lebel S Hould F Julien F Marceau S Bouvet L Long alimentary limb duodenal switch (LADS): a short-term prospective randomized trial. Surg Obes Relat Dis. (2018) 14(1):30–7. 10.1016/j.soard.2017.08.028 29217129

[20] Elias K Hedberg J Sundbom M. Prevalence and impact of acid-related symptoms and diarrhea in patients undergoing Roux-en-Y gastric bypass, sleeve gastrectomy, and biliopancreatic diversion with duodenal switch. Surg Obes Relat Dis. (2020) 16(4):520–7. 10.1016/j.soard.2019.12.020 32057678

[21] Sovik TT Taha O Aasheim ET Engstrom M Kristinsson J Bjorkman S Randomized clinical trial of laparoscopic gastric bypass versus laparoscopic duodenal switch for superobesity. Br J Surg. (2010) 97(2):160–6. 10.1002/bjs.6802 20035530

[22] Pérez N Baltasar A Serra C Ferri L Bou R Bengochea M. Comparative analysis of vertical banded gastroplasty and duodenal switch at five years follow-up. Obes Surg. (2005) 15(7):1061–5. 10.1381/0960892054621143 16105408

[23] Ren CJ Patterson E Gagner M. Early results of laparoscopic biliopancreatic diversion with duodenal switch: a case series of 40 consecutive patients. Obes Surg. (2000) 10(6):514–24. 10.1381/096089200321593715 11175958

[24] Moon RC Kirkpatrick V Gaskins L Teixeira AF Jawad MA. Safety and effectiveness of single- versus double-anastomosis duodenal switch at a single institution. Surg Obes Relat Dis. (2019) 15(2):245–52. 10.1016/j.soard.2018.11.004 30606470

[25] Cottam A Cottam D Portenier D Zaveri H Surve A Cottam S A matched cohort analysis of stomach intestinal pylorus saving (SIPS) surgery versus biliopancreatic diversion with duodenal switch with two-year follow-up. Obes Surg. (2017) 27(2):454–61. 10.1007/s11695-016-2341-7 27568033

[26] Strain GW Torghabeh MH Gagner M Ebel F Dakin GF Abelson JS The impact of biliopancreatic diversion with duodenal switch (BPD/DS) over 9 years. Obes Surg. (2017) 27(3):787–94. 10.1007/s11695-016-2371-1 27686233

[27] Topart P Becouarn G Finel JB. Is transit bipartition a better alternative to biliopancreatic diversion with duodenal switch for superobesity? Comparison of the early results of both procedures. Surg Obes Relat Dis. (2020) 16(4):497–502. 10.1016/j.soard.2019.12.019 32001205

[28] Cottam S Ng P Sharp L Medlin W Cottam DR. Single-anastomosis duodenal ileostomy with sleeve is a safe and effective option for patients in an ambulatory surgical center. Surg Obes Relat Dis. (2019) 15(11):1990–3. 10.1016/j.soard.2019.09.062 31653570

[29] Yashkov Y Bordan N Torres A Malykhina A Bekuzarov D. SADI-S 250 vs Roux-en-Y duodenal switch (RY-DS): results of 5-year observational study. Obes Surg. (2021) 31(2):570–9. 10.1007/s11695-020-05031-z 33047290

[30] Surve A Zaveri H Cottam D Belnap L Cottam A Cottam S. A retrospective comparison of biliopancreatic diversion with duodenal switch with single anastomosis duodenal switch (SIPS-stomach intestinal pylorus sparing surgery) at a single institution with two year follow-up. Surg Obes Relat Dis. (2017) 13(3):415–22. 10.1016/j.soard.2016.11.020 28089438

[31] Pereira AM Guimarães M Pereira SS Ferreira de Almeida R Monteiro MP Nora M. Single and dual anastomosis duodenal switch for obesity treatment: a single-center experience. Surg Obes Relat Dis. (2021) 17(1):12–9. 10.1016/j.soard.2020.09.029 33127321

[32] Tustumi F Bernardo WM Santo MA Cecconello I. Cholecystectomy in patients submitted to bariatric procedure: a systematic review and meta-analysis. Obes Surg. (2018) 28(10):3312–20. 10.1007/s11695-018-3443-1 30097898

[33] Bardaro SJ Gagner M Consten E Inabnet WB Herron D Dakin G Routine cholecystectomy during laparoscopic biliopancreatic diversion with duodenal switch is not necessary. Surg Obes Relat Dis. (2007) 3(5):549–53. 10.1016/j.soard.2007.05.007 17903779

[34] Sucandy I Abulfaraj M Naglak M Antanavicius G. Risk of biliary events after selective cholecystectomy during biliopancreatic diversion with duodenal switch. Obes Surg. (2016) 26(3):531–7. 10.1007/s11695-015-1786-4 26156307

[35] van Vollenstee FA van der Merwe MT. Evaluating the pharmacoeconomic impact of nutrient supplementation post-operatively on patients receiving Roux-Y gastric bypass vs. biliopancreatic diversion with duodenal switch. Obes Surg. (2021) 31(6):2434–43. 10.1007/s11695-021-05268-2 33730334 PMC8113208

[36] Magee CJ Barry J Brocklehurst J Javed S Macadam R Kerrigan DD. Outcome of laparoscopic duodenal switch for morbid obesity. Br J Surg. (2011) 98(1):79–84. 10.1002/bjs.7291 20967827

[37] Strain GW Torghabeh MH Gagner M Ebel F Dakin GF Connolly D Nutrient status 9 years after biliopancreatic diversion with duodenal switch (BPD/DS): an observational study. Obes Surg. (2017) 27(7):1709–18. 10.1007/s11695-017-2560-6 28155056

[38] Topart P Becouarn G Delarue J. Weight loss and nutritional outcomes 10 years after biliopancreatic diversion with duodenal switch. Obes Surg. (2017) 27(7):1645–50. 10.1007/s11695-016-2537-x 28050789

[39] Ballesteros-Pomar MD Gonzalez de Francisco T Urioste-Fondo A Gonzalez-Herraez L Calleja-Fernandez A Vidal-Casariego A Biliopancreatic diversion for severe obesity: long-term effectiveness and nutritional complications. Obes Surg. (2016) 26(1):38–44. 10.1007/s11695-015-1719-2 25982805

